# Putative synaptic genes defined from a *Drosophila* whole body developmental transcriptome by a machine learning approach

**DOI:** 10.1186/s12864-015-1888-3

**Published:** 2015-09-15

**Authors:** Flavio Pazos Obregón, Cecilia Papalardo, Sebastián Castro, Gustavo Guerberoff, Rafael Cantera

**Affiliations:** Departamento de Biología del Neurodesarrollo, Instituto de Investigaciones Biológicas Clemente Estable, Avenida Italia 3318, PC 11600 Montevideo, Uruguay; Instituto de Matemática y Estadística “Prof. Ing. Rafael Laguardia”, Facultad de Ingeniería, Universidad de la República, Montevideo, Uruguay; Zoology Department, Stockholm University, Stockholm, Sweden

**Keywords:** Synapse, Machine learning, Temporal transcription profiles

## Abstract

**Background:**

Assembly and function of neuronal synapses require the coordinated expression of a yet undetermined set of genes. Although roughly a thousand genes are expected to be important for this function in *Drosophila melanogaster*, just a few hundreds of them are known so far.

**Results:**

In this work we trained three learning algorithms to predict a “synaptic function” for genes of *Drosophila *using data from a whole-body developmental transcriptome published by others. Using statistical and biological criteria to analyze and combine the predictions, we obtained a gene catalogue that is highly enriched in genes of relevance for *Drosophila *synapse assembly and function but still not recognized as such.

**Conclusions:**

The utility of our approach is that it reduces the number of genes to be tested through hypothesis-driven experimentation.

**Electronic supplementary material:**

The online version of this article (doi:10.1186/s12864-015-1888-3) contains supplementary material, which is available to authorized users.

## Background

Neuronal synapses are specialized contacts through which neurons communicate with each other or with other cells and are thus fundamental for our understanding of nervous system function. The assembly, function, plasticity and maintenance of synapses require the coordinated expression of a yet undetermined set of genes, which for simplicity we call here “synaptic genes”. Hundreds of synaptic genes have been identified, but there is a broad consensus that these are only a fraction of the total number [1–4] and diverse approaches should be tested to advance in this direction. The basic framework of synapse organization was completed early in metazoan evolution [5–7]. A high degree of conservation among synaptic genes have been found, suggesting relatively little diversification among proteins important for synaptic transmission and demonstrating that knowledge obtained from studies in model organisms is also relevant for other species, including humans [5, 8].

The traditional method to assign a function to a gene involves extensive genetic and biochemical analyses. The generation of catalogues composed of genes with a high probability of having the function of interest narrows this search, thus saving time and resources. New methods for the prediction of gene function have become available with the completion of genome projects and the boom of microarray experiments and predicting gene function is one of the main goals in systems biology and functional genomics [9–11]. As overwhelming volumes of information begun to be stored in large-scale datasets, automatic learning methods emerged as a good strategy to predict gene function. The analysis of microarray expression data through automatic learning methods showed that genes with similar function frequently display similar expression patterns. This suggests a functional relationship among genes whose expression fluctuates in parallel [12–15]. This correlation between biological function and expression pattern suggests that machine learning algorithms could be successfully applied to predict function from expression data [16–20].

Automatic learning methods can be divided into unsupervised and supervised learning. A supervised method “learns” the distinctive features of a given biological function from a training set of genes, in which some of them are known to have the function of interest and others are supposed not to have it. The learned definition is called a “classifier”, and is then used to decide whether or not a gene still not associated with that biological function may have it. Importantly, the predictive quality of the classifier can be objectively estimated in several ways [16].

There is no universally best learning algorithm since even those with the best average performance in a variety of studies perform poorly on other problems or metrics [21, 22]. Ensemble techniques, in which a set of different predictive models is constructed and then their predictions are combined in different ways, have been shown to improve the classification performance [10, 23]. Here we reached very good results applying a very simple ensemble technique by intersecting the classifications of three methods, K- Nearest Neighbors [24], Random Forest [25] and Support Vector Machine [26], that are widely used and are among those with the best average performance when applied to biological data [21, 22]. The predictive quality of a classification task also depends strongly on the training set. We carefully selected the positive samples among those *Drosophila* genes for which a synaptic function was already well demonstrated by independent methods, and the negative samples using clear biological criteria (see Methods).

The fly *Drosophila melanogaster* is one of the model organisms that have contributed the most to our understanding of synapses, for which many synapse genes are already identified through experimental studies [3, 27, 28] and has one of the best annotated genomes [29]. It has an additional advantage for the aim of this study: along its life cycle there are two periods of massive synapse formation [30]. Regarding the quality of the input data, next-generation sequencing technologies (NGST) have overcome some important limitations of microarray technologies, as for example their relatively high rates of false positives and their low accuracy in measurements of transcripts present in low abundance [31]. As input data we used the developmental transcriptome of *Drosophila* published by the MODENCODE Project [32]. This data set, was generated with NGST and has been successfully used for several investigations [33–36]. It appears to be the best available for our objective because it comprises samples corresponding to a period in which there are no synapses, samples corresponding to the two phases of massive and intense synapse formation and samples corresponding to a phase of massive synapse disassembly [30].

A final issue to consider in this type of studies is the estimated number of genes predicted to have the biological function under scrutiny. A synaptic function has been assigned to many *Drosophila* genes by means of genetic screens [37–46] and other experimental approaches (see for example [27]). The proteomic analysis of cellular fractions enriched in synapses has also resulted in catalogues of proteins with a strong probability of being necessary for the synapse. Technical difficulties have so far hampered this option in the fly, but the development of a method adapted to *Drosophila* synaptosomes [47] indicates that this approach will soon lead to the discovery of new synaptic genes. Catalogues of synaptic genes including annotations based on proteomic studies of synaptic components, sequence homology across animal species, protein domains and other bioinformatic approaches such as Gene Ontology [48], SynDB [49] and SynaptomeDB [50] range from a few hundreds to a few thousands genes. By May 2015, SynaptomeDB [50] listed a total of 1886 genes for human synapses and SynDB [49] listed 1073 genes for *Drosophila* synapses and 3249 genes for human synapses. At the beginning of this study, 350 genes were associated with some degree of experimental support to at least one synapse-related Gene Ontology Biological Process (GO BP) in *Drosophila*. Thus, according to a conservative view of the available data, the expected number of synaptic genes in *Drosophila* could be set around 1000 for the purpose of the approach tested here.

The aim of this study was to test whether catalogues enriched in genes with a certain biological function can be generated solely from transcription data with a group of supervised learning algorithms. Our objective was to obtain a catalogue of *Drosophila* genes with high probability of having a synaptic function. The approach was fruitful and we present a catalogue that according to several biological criteria appears to be greatly enriched in new synaptic genes.

## Results

### Pre-processing of the original dataset

**Fig. 1:**
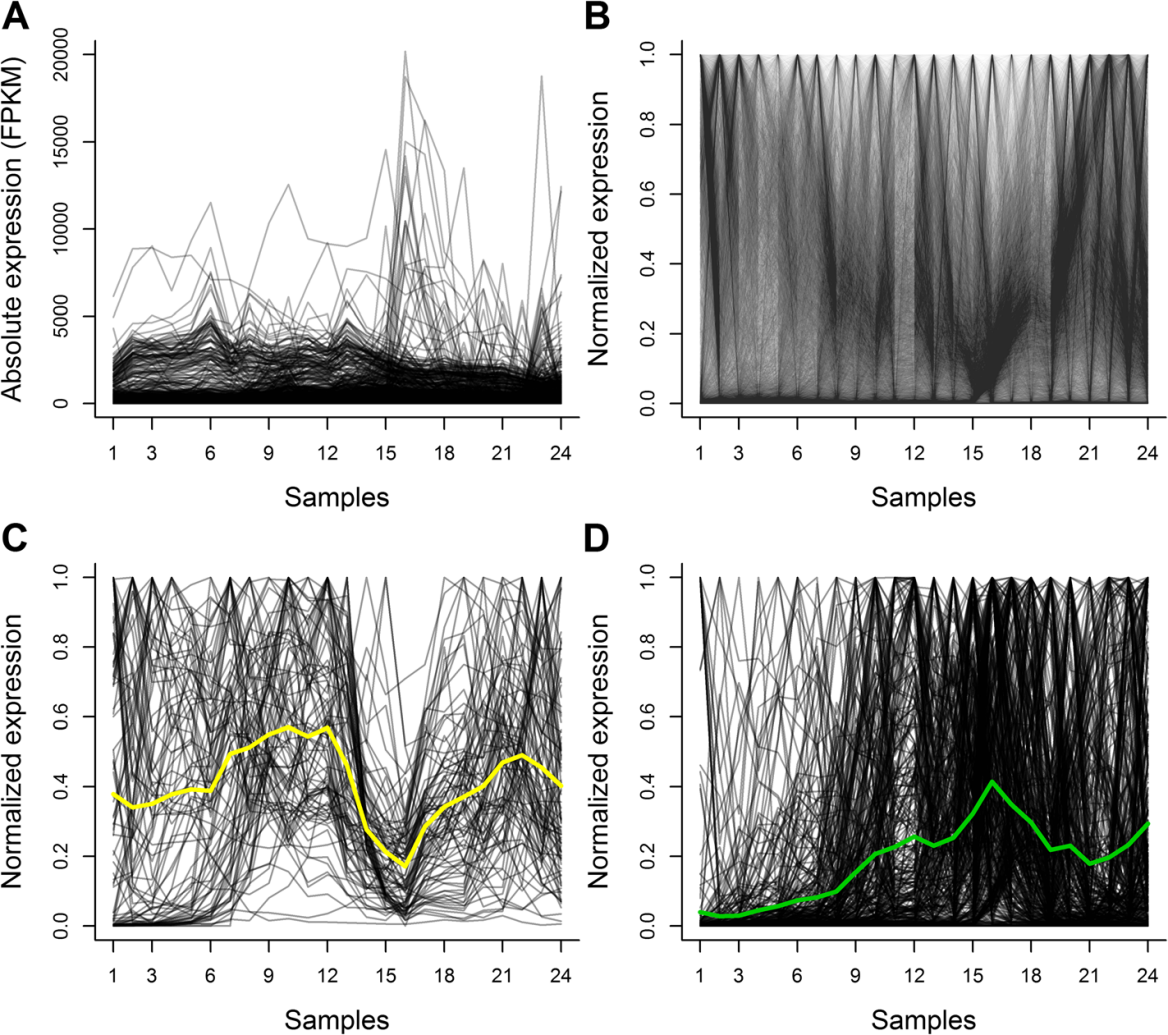
Transcription profiles of the *Drosophila* genes to be classified and of the training set. **a**–**b** Temporal expression profiles of the 13,642 *Drosophila* genes that show transcription level above zero in at least one sample during embryonic, larval or pupal stages. Graphs were constructed with absolute values (FPKM, **a**) and with the same values after normalization between 0 and 1 (**b**, see Methods). **c** Transcription profiles of the 92 “synaptic genes” of the training set after normalization of the original values between 1 and 0. A clear correspondence is observed between what is expected for the mean transcription profile of Drosophila synaptic genes and the actual expression profiles of the genes of our training set. Along the life cycle of *Drosophila* a first wave of massive synaptogenesis takes place during the second half of embryonic life (samples 7–12), and a second wave occurs in the pupa (samples 19–24), when the synapses of the adult brain are being formed. Between these two waves of synaptogenesis a period of massive synapse disassembly takes place. The yellow line corresponds to the mean expression levels of the 92 synaptic genes and matches these three features. **d** Transcription profiles of the 397 “non-synaptic genes” of the training set, after normalization of the original values between 1 and 0. The green line corresponds to the mean expression levels of the 397 non-synaptic genes. (Original values published by Graveley et al. [32] and adapted as explained in Methods)

We used the developmental transcriptome of *Drosophila melanogaster* published by the MODENCODE Project [32], but we considered only the 13,642 genes that showed transcript levels above zero at least in one of the 24 samples corresponding to the embryo, the larva and the pupa (see Methods). The absolute and the normalized transcription profiles of these 13,642 genes are shown in Fig. 1a and b respectively. We then used 489 of these genes to train our algorithms and classify the remaining 13,153 genes into either “synaptic genes” or “non synaptic genes”.

### Construction of the training set

To train our classifiers we defined the labels “synaptic genes” and “non-synaptic genes”. The 92 genes that we labeled as “synaptic genes” (see Methods) and the corresponding bibliographic references are presented in Additional file 1. The corresponding normalized transcription profiles are shown in Fig. 1c. The 397 genes that fulfill at least one of the two biological criteria defined to label a gene as “non-synaptic” (see Methods) are shown in Additional file 2 and Fig. 1d shows their normalized transcription profiles.

### Model adjustment

**Fig. 2:**
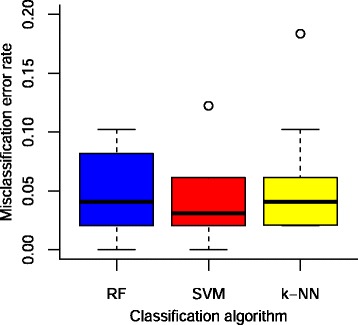
Misclassification error rates of the three adjusted models after 10-fold cross validation. Box plots of misclassification error rates of the three adjusted models: Random Forest (RF), Support Vector Machine (SVM) and k-Nearest Neighbors (k-NN), estimated by 10-fold cross validation as described in the text. In each box plot the black horizontal line represents the median value and the points outside the box correspond to values over or lower to 1.5 times interquartile range than the third or first quartile respectively

The mean error and dispersion for the three adjusted models after 10-fold cross validation over the training set are shown in Fig. 2. The three classifiers reached error rates below 5 %. For the details on the adjustment of each model see Methods. The area under the ROC curve of each classifier reached values above 0.97 in the three cases (Additional file 3).

### Initial classification of the three models

**Fig. 3:**
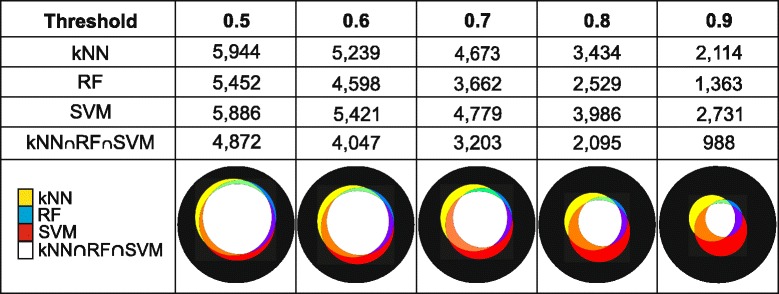
Number of genes classified as synaptic by each method as the classification threshold increases. Each column corresponds to the threshold that a gene’s estimated probability of being synaptic must exceed to be labeled as synaptic. Each row corresponds to one of the adjusted models or to one of their combinations. The last row shows the number of genes classified as synaptic by the three models. In the bottom panel the color areas of the Venn diagrams are proportional to the number of genes that they represent. The 13,153 genes to be classified are represented by the black circles and the number of genes classified as synaptic by each model, or combinations thereof, are represented in agreement with the color code shown to the left

After adjusting the three models we classified our dataset and obtained three initial catalogues of putative synaptic genes (Fig. 3). The three classifications display a high degree of coincidence, with k-NN, the model that produces the more divergent catalogue of genes, showing more than 83 % of coincidence with the other two models. The genes classified as synaptic by the three models represent a consensus catalogue of 4872 genes. This initial consensus catalogue is much bigger than what is expected for a catalogue of synaptic genes, as discussed in the Background section.

### Sequential increase of the classification threshold

To reduce the size of the initial consensus catalogue, as well as to improve its statistical strength, we sequentially increased the classification threshold, excluding those genes whose classifications have higher probabilities of being false positives (see Methods). This procedure generates a series of catalogues of gradually decreasing size for which a biological characterization done *a posteriori* can provide additional support. The number of genes classified as synaptic by each model as the threshold increases, as well as the degree of coincidence between the classifications are shown in columns 2 to 5 of Fig. 3. The increment of the classification threshold was associated with an increment of the global error rate (Additional file 4).

### Functional enrichment analysis

**Fig. 4:**
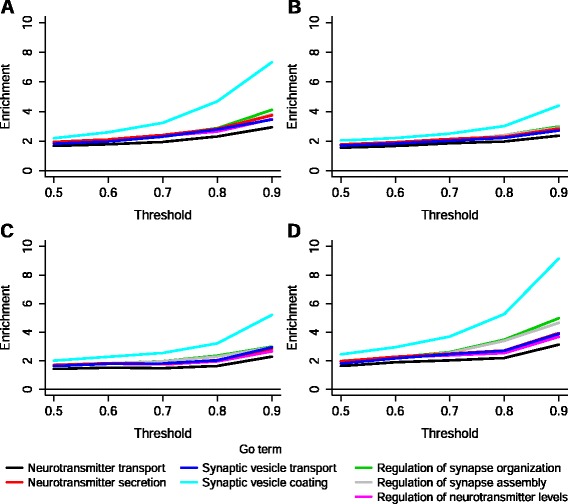
Enrichment in selected synapse-related BP GO terms as the classification threshold increases. **a**–**d** show the enrichment of the catalogues generated by each classifier, in a representative selection of GO terms of relevance for the biological function “synapse”. For all classifiers, elevation of the threshold resulted in increased enrichment. This increase in functional enrichment is accentuated in the consensus catalogues, showing how the combined methods improved the three individual classifiers. **a** k-NN catalogues, **b** Random Forest catalogues, **c** SVM catalogues, **d** catalogues of genes classified as synaptic by the three classifiers. All enrichment values have an associated p-value lower than 10^−4^ and a FDR q-value lower than 10^−3^

We evaluated the functional enrichment of our catalogues in synapse-related GO BP terms. All our catalogues were enriched in several of these terms, and the degree of enrichment increased with the increase of the classification threshold. This direct relationship between threshold and enrichment is illustrated in Fig. 4, which shows the enrichment in a representative selection of synapse-related GO BP terms found in the series of catalogues produced by each of the three learning algorithms as well as in the corresponding consensus catalogues.

### Genes with Tissue-Specific Differential Expression (GTSDEs)

**Fig. 5:**
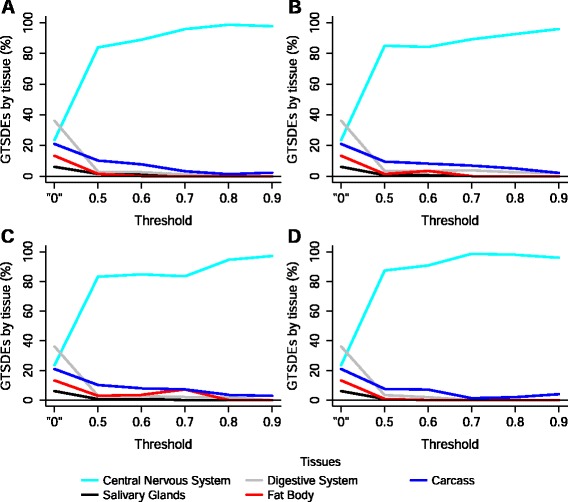
Relation between percentages of genes with tissue-specific differential expression by tissue and classification thresholds. **a**–**d** show the relationship between tissue-specific differential expression, classification methods, and classification thresholds. Regardless of the classification method, all catalogues are enriched in genes expressed in the CNS at much higher levels than in tissues with fewer or none synapses. Notice that increasing the threshold did not result in an increment of the proportion of genes differentially expressed in tissues other than the CNS. **a** k-NN catalogues, **b** Random Forest catalogues, **c** SVM catalogues, **d** consensus catalogues. The threshold values are shown in the horizontal axis, with 0 corresponding to the initial set of genes to be classified. Each colored line corresponds to one of the five analyzed tissues according to the color code shown at the bottom

Among the 13,153 genes committed to the classifiers, there are 686 that have tissue-specific differential expression (as defined in Methods, complete list is provided as Additional file 5). The percentage of GTSDEs we found in each tissue is roughly 24 % for the Central Nervous System (CNS), 4 % for the salivary glands, 12 % for the fat body, 37 % for the digestive system and 23 % for the carcass. Since the overwhelming majority of synapses are formed by neurons inside the CNS, a catalogue of synaptic genes is expected to have an over-representation of GTSDEs in this tissue. On the other hand, we expect the GTSDEs in the salivary glands or fat body (tissues without synapses) to be under-represented in our catalogues. The results of this test are shown in Fig. 5. In all the catalogues there is a great over-representation of GTSDEs in the CNS. This enrichment increases with higher threshold. At the same time, the enrichment in GTSDEs in those tissues without synapses (the salivary gland and the fat body) or in tissues with much fewer synapses (digestive system and carcass) decreases.

### Final catalogue of 893 putative *Drosophila* synaptic genes

Our consensus catalogue corresponding to a classification threshold of 0.9 comprises 988 *Drosophila* genes that, according to the three adjusted algorithms, have a probability above 90 % of being involved in synapse assembly and function. Among all the catalogues obtained in this study, this is the one showing the highest enrichment in synapse-related GO BP terms, as well as the highest proportion of GTSDE in the CNS. The functional enrichment of this catalogue is explained by the presence of 95 genes that were already annotated as relevant for synapse assembly or function in the GO database by July 2014. Excluding these genes, we obtain our final catalogue of 893 putative synaptic genes. This catalogue is provided as Additional file 6. Additional file 7 contains the predicted probability of being synaptic for all the genes in the data set and Additional file 8 shows the transcription profiles of the genes classified as synaptic at each threshold.

### Genes of our final catalogue have human homologues already described as synaptic genes

We found that 607 genes of the 893 in our final catalogue have at least one homologue in *Homo sapiens* [51]. Of these 607 *Drosophila* genes, 11 % (66 genes) have a human homologue with a synapse related annotation [52]. This is relevant because these human synaptic genes are homologues of *Drosophila* genes belonging to a catalogue from which any gene already annotated as synaptic was selectively excluded. The FlyBase IDs and the symbols of these 66 genes, as well as their human homologues with their corresponding synapse-related GO annotations are shown in Table 1.

**Table 1:** Putative synaptic genes with no synapse-related annotation in *Drosophila* and homologues annotated as synaptic *in Homo sapiens*

Annotation symbol	Gene symbol	Mean prob.	Human homologue	HGI	GO term (in Homo sapiens)
CG7392	Cka	1.00	STRN	ENSG00000115808	CC: postsynaptic membrane
CG9634	NA	1.00	MME	ENSG00000196549	CC: synaptic vesicle
CG8529	Dyb	0.99	DTNA	ENSG00000134769	BP: synaptic transmission
CG7023	Usp12–46	0.99	USP46	ENSG00000109189	BP: regulation of synaptic transmission
CG16973	msn	0.99	MINK1	ENSG00000141503	BP: synaptic transmission
CG6593	Pp1alpha-96A	0.99	PPP1CA	ENSG00000172531	CC: dendritic spine
CG15112	ena	0.99	ENAH	ENSG00000154380	CC: synapse
CG10545	Gbeta13F	0.99	GNB2	ENSG00000172354	BP: synaptic transmission
CG14991	Fit1	0.99	FERMT1	ENSG00000101311	CC: synapse
CG1651	Ank	0.99	ANK2	ENSG00000145362	CC: postsynaptic membrane
CG32717	sdt	0.99	MPP4	ENSG00000082126	CC: presynaptic membrane
CG5248	loco	0.99	RGS14	ENSG00000169220	BP: long-term synaptic potentiation
CG7147	kuz	0.99	ADAM10	ENSG00000137845	CC: postsynaptic density
CG10566	NA	0.99	ICA1	ENSG00000003147	CC: synaptic vesicle membrane
CG32264	NA	0.99	PHACTR1	ENSG00000112137	CC: synapse
CG13830	NA	0.99	SPOCK2	ENSG00000107742	BP: synapse assembly
CG10637	Nak	0.98	AAK1	ENSG00000115977	CC: terminal bouton (of the axon)
CG3269	Rab2	0.98	RAB14	ENSG00000119396	BP: neurotransmitter secretion
CG10011	NA	0.98	ANK2	ENSG00000145362	CC: postsynaptic membrane
CG8440	Lis-1	0.98	PAFAH1B1	ENSG00000007168	BP: synaptic transmission
CG5650	Pp1-87B	0.98	PPP1CA	ENSG00000172531	CC: dendritic spine
CG10579	Eip63E	0.98	CDK16	ENSG00000102225	CC: synaptic vesicle
CG10538	CdGAPr	0.98	ARHGAP32	ENSG00000134909	CC: postsynaptic membrane
CG7535	GluClalpha	0.98	CHRNA5	ENSG00000169684	BP: synaptic transmission
CG8726	NA	0.98	PXK	ENSG00000168297	BP: regulation of synaptic transmission
CG30389	NA	0.98	TMEM57	ENSG00000204178	CC: synapse part
CG7546	NA	0.98	BAG6	ENSG00000204463	BP: synaptonemal complex assembly
CG1506	Ac3	0.98	ADCY3	ENSG00000138031	BP: synaptic transmission
CG6214	MRP	0.98	ABCC8	ENSG00000006071	BP: synaptic transmission
CG4574	Plc21C	0.98	PLCB1	ENSG00000182621	BP: synaptic transmission
CG11734	HERC2	0.98	HERC1	ENSG00000103657	MF: neurotrans:Na symporter activity
CG42829	CadN2	0.97	CDH2	ENSG00000170558	CC: synapse
CG6383	crb	0.97	DNER	ENSG00000187957	BP: synapse assembly
CG1862	Ephrin	0.97	EFNB1	ENSG00000090776	CC: synapse
CG7100	Cadherin-N	0.97	CDH1	ENSG00000039068	BP: synapse assembly
CG8948	Graf	0.97	OPHN1	ENSG00000079482	BP: synaptic vesicle endocytosis
CG9361	Task7	0.97	KCNK3	ENSG00000171303	BP: synaptic transmission
CG6998	ctp	0.97	DYNLL2	ENSG00000121083	BP: synaptic target recognition
CG18455	Optix	0.97	SIX1	ENSG00000126778	BP: reg. of synaptic growth at nj
CG5912	arr	0.96	LRP6	ENSG00000070018	BP: synaptic transmission CC: synapse
CG8261	Ggamma1	0.96	GNG10	ENSG00000242616	BP: synaptic transmission
CG2849	Rala	0.96	RIT2	ENSG00000152214	BP: synaptic transmission
CG32217	Su(Tpl)	0.96	MARVELD2	ENSG00000152939	MF: neurotrans:Na symporter activity
CG17336	Lcch3	0.96	GABRB1	ENSG00000163288	BP: synaptic transmission
CG15274	GABA-B-R1	0.96	GABBR1	ENSG00000204681	BP: synaptic transmission
CG4244	Su(dx)	0.96	NEDD4	ENSG00000069869	BP: regulation of synapse organization
CG16757	Spn	0.96	PPP1R9A	ENSG00000158528	CC: synapse
CG4625	Dhap-at	0.96	GNPAT	ENSG00000116906	BP: synapse assembly
CG32434	siz	0.96	IQSEC3	ENSG00000120645	CC: inhibitory synapse
CG9491	Gef26	0.95	RAPGEF2	ENSG00000109756	BP: regulation of synaptic plasticity
CG42314	PMCA	0.95	ATP2B2	ENSG00000157087	BP: regulation of synaptic plasticity
CG7223	htl	0.95	FGFR2	ENSG00000066468	BP: synaptic vesicle transport
CG9375	Ras 85D	0.95	HRAS	ENSG00000174775	BP: long-term synaptic plasticity
CG30388	Magi	0.95	MAGI2	ENSG00000187391	CC: synapse
CG11958	Cnx99A	0.95	CANX	ENSG00000127022	BP: synaptic vesicle endocytosis
CG9985	sktl	0.94	PIP5K1C	ENSG00000186111	BP: synaptic vesicle exo and endocitosis
CG8726	NA	0.94	KCNK18	ENSG00000186795	BP: synaptic transmission
CG7641	Nca	0.94	NCALD	ENSG00000104490	BP. synaptic transmission
CG8394	VGAT	0.94	SLC32A1	ENSG00000101438	BP: synaptic transmission
CG3585	Rbcn-3A	0.94	DMXL2	ENSG00000104093	CC: synaptic vesicle
CG7558	Arp3	0.94	ACTR3	ENSG00000115091	CC: excitatory synapse
CG14145	Blos2	0.94	BLOC1S2	ENSG00000196072	BP: synaptic vesicle transport
CG1407	NA	0.94	ZDHHC15	ENSG00000102383	BP: synaptic vesicle maturation
CG31196	14-3-3epsilon	0.94	YWHAE	ENSG00000128245	BP: regulation of synaptic plasticity
CG16928	mre11	0.94	MRE11A	ENSG00000020922	CC: synapsis
CG8705	pnut	0.92	SEPT5	ENSG00000184702	BP: synaptic vesicle targeting

The table shows the list of *Drosophila* genes belonging to our final catalogue of putative synaptic genes that don’t have any synapse related GO term annotation but have at least 1 homologue in *Homo sapiens* already annotated with some synapse related GO term. The first 2 columns show the Annotation Symbol as well as the Symbol of these 66 *Drosophila* genes. The third column shows the mean between the classification probabilities given by the three learning algorithms to each gene. The following columns show the symbol and gene identifier of the corresponding human homologue (or 1 of them when there are more than 1) that is already annotated with at least 1 synapse related GO term in humans, an annotation that is shown in the last column of the table

As a way to estimate the statistical relevance of this result, one should construct a list of 988 *Drosophila* genes randomly selected from the initial set of genes to be classified, and then determine (after excluding from the list all the genes with any synapse related annotation), how many genes have at least one human homologue with any synapse related annotation. After repeating this procedure three different times, we obtained a much lower number of genes (25 genes in each case). This result reinforces the conclusion that our final catalogue is enriched in genes of importance to the synapse in both species.

### Comparison with a list of rat synaptic proteins

In a recently published paper [53], Wilhelm and collaborators selected a set of rat proteins for which localization in the synapse had been well established. As their aim was to obtain a three-dimensional reconstruction of the synapse, the list includes not only the kind of proteins that we have defined as “synaptic” in our study, but also more ubiquitous proteins. As an additional way to evaluate our results, we “translated” [51, 52] Wilhelm’s list of rat proteins into a list of *Drosophila* “synaptic genes”. After excluding those genes not fulfilling our definition of synaptic genes (as for example cytoskeletal and mitochondrial proteins) or without homologues in *Drosophila,* we obtained a list of 53 *Drosophila* genes, whose rat homologues have a well established synaptic function.

We found that 14 of these 53 genes had been included in our training set and 28 of the remaining genes were already annotated with at least one synapse related GO term in Drosophila (and so they were selectively excluded from the data set we were classifying). There are then 11 genes in Wilhelm’s list that are neither in our training set nor already annotated as synaptic genes in *Drosophila*. If there is good conservation between mammals and *Drosophila* synaptic proteins one could expect our classifiers to label as synaptic genes at least some of these 11 genes. This turned to be the case, as 9 of these 11 genes belong to our initial consensus catalogue and 5 belong to our final catalogue of putative synaptic genes. We believe these results (showed in Table 2) reinforce the reliability of our approach.

**Table 2:** Genes in Wilhelm et al. [53] list that are neither in our training set nor annotated as synaptic in *Drosophila*

Rat protein	Drosophila homologue	Initial catalogue	Final catalogue
VGlut 1/2	CG10069	Yes	Yes
Calmodulin	CG8472	Yes	No
NSF	CG31495	Yes	No
AP-2 mu2	CG10637	Yes	Yes
SGIP1	CG8176	Yes	Yes
endophilin II	CG9834	Yes	Yes
Hsc70	CG8937	No	No
PIPK Ig	CG9985	Yes	Yes
Vti1a	CG3279	Yes	No
VAMP4	CG1599	Yes	No
Calbindin	CG6702	No	No

The first column shows the names of the rat proteins whose *Drosophila* homologues are neither in our training set nor already annotated as synaptic genes in the fly. The second column shows the name of the corresponding *Drosophila* homologue. The third column indicates whether these *Drosophila* genes were classified as synaptic with a probability higher than 0.5 by the 3 algorithms. The fourth column indicates if the genes were also classified as synaptic by the 3 algorithms with a probability above 0.9

## Discussion

One of the main goals of functional genomics is to make predictions on the biological function of genes from the information stored in large-scale datasets, maximizing the utility of available information and making predictions of function with verifiable performance [11]. Here we show that it is possible to obtain gene catalogues enriched in genes of importance for the neuronal synapse, analizing a genome-wide full-body developmental transcriptome with a combination of supervised learning algorithms and a bioinformatic approach tested here for the first time.

Some 25 years ago, a functional relationship between genes displaying similar expression patterns was suggested by the first studies analyzing expression data with automatic learning methods. Clustering of yeast genes on the basis of their similarities in expression patterns resulted in groups of genes sharing important functional similarities [11, 13]. A correlation between expression profile and biological function was also demonstrated in *Drosophila* [54–57] and humans, although in this last case somewhat obscured by a less complete functional annotation of the genome [12]. On the other side, a clear mapping of functional gene groups to expression profiles was demonstrated in rats [14]. Since then, the use of automatic learning methods to assign putative functions to genes, based not only on their expression profiles but also on protein—protein interactions and on structural similarities, has led to a broad diversity of strategies and to a profuse bibliography [16–20, 58].

The most direct antecedent to our study is, we believe, that of Yan and collaborators [11]. In that work, function-specific classifiers based on Random Forest were trained to predict GO terms for *Drosophila melanogaster* genes using features from protein-protein interaction networks, gene expression profiles, genetic interactions, conserved protein domains and cross-species sequences similarities. Regarding synapse assembly and function, GO terms including the words “synapse”, “synaptic” or “neurotransmitter” were predicted for only 31 genes [11].

Our study differs from this and other previous studies in several important issues. As far as we know it is the first to apply automatic learning algorithms to predict gene function using developmental transcriptome data obtained through NGST. The developmental transcriptome used here has several advantages over other available *Drosophila* RNAseq data sets. Since the formation of the brain in the fly embryo is such a rapid process and the transcription profiles show good temporal correlations with the sequence of biological processes [59], this sample set offers a clear advantage for the definition of putative synaptic genes. The key feature that made this data set the best available for our study is that it spans over several stages of the life cycle relevant for the classification. These include a stage in which there are no synapses in the organism, two stages when massive, intense synapse formation occurs, and a stage of massive synapse disassembly, all of which improves the potential of the algorithms to distinguish between synaptic and non-synaptic genes. This ability to discern synaptic genes produced a catalogue not only of the sought size, but whose functional enrichment in synapse related GO terms is higher and more diverse than the enrichment found in a list of genes with CNS-specific differential expression (data not shown).

Another novelty of our study is that the training set was constructed to be substantially different from the set of genes that are already annotated in the GO database with some synapse-related GO term. This strategy avoids any circularity problem in evaluating the resulting catalogues by analyzing their functional enrichment in synapse-related GO terms. Since the *Drosophila* genome is one of the best annotated [60, 61], this represents a great advantage over studies in which the performance of the classifiers is evaluated only by cross-validation over the training set or by literature examination of the top scoring predictions. It is important to notice here that our training set of synaptic genes only includes genes for which their importance for the formation and function of synaptic active sites had been demonstrated with independent methods. We did not include genes with more general annotations (as for instance “axon” or “neuromuscular junction”) to render our analysis more neutral to potential differences related to variation among organisms in the morphology of their dendrites and axon terminals.

Another important feature of our study is that we followed a procedure that improves the classification performance [10, 23], training different learning algorithms and taking a vote over their predictions. The advantage of this approach is illustrated by the fact that for a given threshold, the functional enrichment in synapse-related GO terms of the consensus catalogue is bigger than that of the catalogues corresponding to each algorithm. This procedure has the additional advantage of reducing the probability of including false positives in our final catalogue. Since our aim was not the generation of an exhaustive catalogue of synaptic genes, but a catalogue of genes with high probability of being synaptic, a decrease in the number of false positives is preferable, although this leads to an increase in the number of false negatives. It is worth noting the very low error rates reached by the three algorithms in their initial classifications and the excellent quality of their performance, estimated through calculation of the area under the ROC curve.

Finally, we wish to emphasize the fact that 11 % of the *Drosophila* genes in our final catalogue of 893 genes are already known to be of importance for the synapse in humans, although still not in the fly. On the other hand, 9 out of 11 genes already known as being important for synaptic function in rats [53] but not yet in *Drosophila*, were classified as synaptic by our method. We think that these coincidences are explained by the high degree of functional conservation among homologous genes between these species, which makes undoubtedly a very strong argument in favour of the convenience of our approach and of the quality of our final catalogue.

## Conclusions

The strong correlation between classification threshold, functional enrichment and proportion of GTSDE in the CNS, together with the observation that 11 % of the *Drosophila* genes in our final catalogue have human homologues already annotated as synaptic genes in *H. sapiens*, strongly suggest that our final catalogue is highly enriched in genes of relevance for *Drosophila* synapse assembly and function but still not recognized as such.

The utility of our approach is that it reduces the number of genes to be tested through hypothesis-driven experimentation. Thus, we make available the list of 893 putative synaptic genes to the scientific community, firmly believing that this will facilitate their investigation, by means of gene silencing, mutant analysis, behavioral assays and other traditional protocols. This will most likely lead to the identification of new genes of great relevance for the normal function of the nervous system.

## Methods

### Temporal transcriptome

We used the developmental transcriptome of *Drosophila melanogaster* published by the MODENCODE Project [32]. In this data, each sample consisted of total polyAAA-RNA isolated from 30 whole bodies obtained at 27 unequally spaced moments along the organism life cycle, including 12 points during the embryonic stage, 6 points during the larval stage, 6 points during the pupal stage and 3 points during the adult stage (separated by sex). We used the data published by these authors as “Supplementary Table 9”, which shows the transcript levels of the 15,398 genes expressed in this study as fragments per kilo base of exon per million fragments mapped (FPKM). From this initial set of 15,398 genes we excluded 1756 genes that show transcript levels above zero only during adult life, because we were looking for genes expressed in the embryo and in the pupa, two developmental periods where massive synaptogenesis take place [30]. Assuming that profile shapes and temporal correlations with other transcription profiles are generally more informative about the function of a gene than its absolute transcription levels, we normalized each gene’s temporal series dividing it by its maximum value, thus obtaining for each gene a series of values oscillating between 0 and 1. After excluding from the 13,642 remaining genes the 489 genes that were used to construct the training set (see below), we obtained a set of 13,153 genes that were then classified into either “synaptic genes” or “non synaptic genes”.

### Construction of the training set

Gene Ontology (GO) provides a valuable source of structured knowledge of protein functions [48] and has been widely used as a source of positive and negative samples to train classifiers [11, 62, 63]. Typically, all genes associated with the function being studied and annotated as such in the GO database, are included in the training set as positive samples. The negative samples are then randomly selected from all the non-positive samples. This strategy has some important drawbacks. Considering all non-positive samples as negative samples not only can cause a high imbalance between both classes, generating serious model fitting problems [64], but because many genes most probably have functions yet not annotated, this procedure does not minimize the chance of including false negatives in the training set.

After an exhaustive bibliographic revision, we elaborated a list of 92 “synaptic genes” for which a synaptic function is supported by strong and unequivocal experimental evidence. We chose a widely-accepted definition of “synapse” that refers to the specialized cell-to-cell contact also called “active zone”, formed by a pre-synaptic and a post-synaptic zone and separated by a synaptic cleft in which the pre-synaptic zone releases neurotransmitter upon arrival of an action potential [28]. We took special care not to include as “synaptic genes” those genes considered as such solely on the basis of less strict definitions of “synapse”, as for example “synaptic boutons” or “neuromuscular junction” (For this semantic problem, see [28]). Importantly, we left out of the training set the majority of genes that by July 2014 were already annotated with some synaptic function in the GO database. We included only 83 out of the 456 genes that were annotated with at least one synapse-related GO BP term. On the other hand, we included nine genes that, although the strong experimental evidence for their synaptic function, were not yet annotated as such in the GO database. This procedure allowed us to test the functional enrichment of the resulting catalogues against the GO database without any circularity problem.

To elaborate the list of “non-synaptic genes”, we selected genes fulfilling at least one of the two following biological criteria:

- To have a very low expression level in the Central Nervous System (CNS) throughout the third larval instar relative to their mean expression level in the whole body during the same stage. As this is a developmental stage of rapid growth and intense synapse formation, we assumed that these genes will most likely be of little relevance for synapse assembly and function. To determine which genes fulfill this criterion, we used the tissue-specific expression data available in FlyAtlas [65]. We found that 352 genes from our data set showed a ratio lower than 0.05 between their expression in the CNS and their mean expression in the whole body at the third instar larva.
- To have an extreme sex-biased expression, because we expect synapses to be essentially the same in females and males. We analyzed the six samples of the MODENCODE developmental transcriptome that correspond to the adult life, that were prepared and sequenced for males and females separately [32]. We found 45 genes that showed expression levels equal to 0 in one sex and higher than 25 FPKM in the other sex.

We stress the fact that neither the “synaptic genes” nor the “non-synaptic genes” were labeled taking into account the transcription profiles by which the rest of the genome was classified.

### Machine learning approach

We used three well established non-parametric and supervised classification methods to predict whether a gene **i** is synaptic (y_i_ = 1) or not (y_i_ = 0) given its temporal transcription profile {**x**_i_ = x_i_^j^: j = 1, 2,…, 24}. These methods allow to estimate the probabilities **P**(y _i_ = 1| **x**_i_) from a training set. A decision rule is applied and gene **i** is classified as synaptic if the estimated probability is larger than a given threshold, which by default is set at 0.5. To evaluate the goodness of a classifier, the true error rate is estimated by 10-fold cross validation. Each procedure involves two parameters as described below and these parameters modify the threshold over which a given gene is classified as synaptic [66]. All calculations were performed using the R [67] packages *randomForest* [68], *-e1071* [69] and *class* [70].

#### Random forest

This is an aggregation method based on classification trees that improves their results by introducing two randomization steps: a number B of bootstrap samples from the training set and the random selection of m predicting variables. The estimation of **P**(y_i_ = 1| **x**_i_) is obtained by aggregating the predictions of each one of the B trees in the forest [25]. We constructed 500 classification trees and used 4 variables in each partition.

#### Support vector machine

This method searches for the optimal hyper-plane separating the training set according to the label of the data (synaptic or not synaptic, in our case). The margin of separation is maximized subject to a constraint related to the total cost, C, of violating the margin. By using a Radial Basis Kernel [26, 66] we enlarged the feature space to improve the separation; the width of the kernel is controlled by a parameter γ. We adjusted C and γ (being γ the exponent in the radial kernel) by a grid search and the lowest error rate, shown in Fig. 2, was 3.3 % with C = 2.2 and γ =0.02.

#### k-NN

In k-Nearest Neighbors classification an unlabeled object is classified according to the most common class among its labeled ***k*** nearest neighbors. The parameters to adjust are the distance in feature space and ***k*** [24]. Using Euclidean distance and increasing the number of considered neighbors one by one, we observed that after 7 neighbors the error rate estimation by 10-fold cross validation reached a stable value (data not shown). Nevertheless, we decided to use 25 neighbors, as this allowed us to modify more freely the classification threshold to label a gene as “synaptic” (see below). This is the error for k-NN shown in Fig. 2.

### True error rate estimation

To evaluate the goodness of each classifier we performed 10 fold cross validation over the training set. To do so, the training set was randomly split in ten subsets of the same size. Each model was then trained with 9 of the subsets and tested with the remaining one. As the true labels of the training set are known, the error rate of the trained model can be quantified. This procedure is repeated 10 times, leaving out each time 1 of the 10 subsets. The mean error rate is then calculated as an estimation of the true error rate of the classifier. The same method was used to calculate the error rates of the classifiers at different classification thresholds.

### Sequential increase of the classification threshold

Instead of directly setting the classification thresholds values needed to produce a catalogue of the expected size, we decided to sequentially increase the classification threshold, that is, the estimated probability of being a synaptic gene that a gene must exceed to be classified as synaptic. This classification threshold has a default value of 0.5 and we considered the catalogues obtained with classification thresholds of 0.6, 0.7, 0.8 and 0.9. This procedure leads to a decreasing in size series of catalogues formed by genes that are “synaptic genes” according to each of the adjusted model. The procedure also results in a decreasing in size series of consensus catalogues, formed by genes that are “synaptic genes” according to the three adjusted models.

### Biological characterization of the catalogues

To investigate whether the increment of the statistical constraints resulted in an increase of the biological relevance of the catalogues we determined two of their features: the catalogue’s enrichment in synapse-related BP GO terms and the proportion of the catalogue’s genes with a differential expression in the CNS.

### Functional enrichment analysis

To evaluate the biological quality of the gene catalogues produced by our approach, we determined their enrichment in BP GO terms using GOrilla [71]. This GO enrichment analysis tool allows comparing the presence of GO terms in a given gene catalogue with respect to a custom background set of genes. In our case, this background set was composed by the genes that remained after excluding from the initial set of 15,398 genes, the 1756 genes that showed expression only during adult life, and the 489 genes that belong to the training set. That is, we measured the enrichment of each catalogue using as background set the 13,153 genes to be classified. Here, enrichment is defined as **(b/n) / (B/N)**, with **N** being the total number of genes, **B** being the total number of genes associated with a specific GO term, **n** being the number of genes in the target set and **b** being the number of genes in the intersection [71]. We only considered enrichments with an associated p-value lower than 10^−4^ and a FDR q-value lower than 10^−3^. FDR q-value is the correction of the p-value for multiple testing using the Benjamini and Hochberg method.

### Genes with tissue-specific differential expression

As an additional way to evaluate the biological relevance of our gene lists, we analyzed tissue-specific differential expression with the assumption that synaptic genes are expressed at higher levels in the CNS (the tissue with most synapses per unit of volume) than in the salivary glands, intestine or other tissues with none or fewer synapses. To do so, we used the data published by modENCODE and made available by Flybase in http://flybase.org/static_pages/expression/expr_search.html [72]. We selected five tissues to analyze: the CNS, the salivary glands, the fat body, the digestive system and the carcass. The salivary glands and the fat body have no synapses because they are not innervated whereas the intestine and carcass, although innervated, posses much less synapses than the CNS. Using the forms in Flybase we selected the genes that during the third larval instar show at least a moderate expression in one of the five tissues and a very low expression in all the other considered tissues. By this procedure we constructed five lists of genes that are differentially expressed in each of the five selected tissues (See Additional file 4). As we expect a catalogue of synaptic genes to be enriched in genes that are preferentially expressed in the CNS, the five lists of GTSDE can be used to evaluate the quality of our catalogues. We determined the proportion of the differentially expressed genes that do so in each of the considered tissues and focused on how this proportion changes as we increase the classification threshold.

### Genes of our final catalogue with human homologues already annotated as synaptic genes

Our consensus catalogue corresponding to a classification threshold of 0.9 comprises 988 *Drosophila* genes that, according to the three algorithms, have a probability above 90 % of being involved in synapse assembly and function. After excluding those genes that were already annotated as relevant for synapse assembly or function in the GO database (July 2014), we obtained a final catalogue of 893 putative synaptic genes. As a way to further characterize this catalogue, we explored how many of these genes have at least one human homologue with some synapse related annotation [52]. To do so we batch-downloaded the human homologues to our final catalogue from FlyBase, and then we downloaded the GO annotation for those genes from MetabolicMine.

## Additional files

Additional file 1:
**List of synaptic genes and references.** A .csv file listing the 92 genes labeled as “synaptic genes” and the corresponding bibliographic references. (CSV 25 kb) Additional file 2:
**List of non-synaptic genes.** A .csv file listing the 397 “non-synaptic” genes. (CSV 10 kb) Additional file 3:
**ROC curves of the three implemented classifiers.** A .pdf file with the ROC curves of the three classifiers. (PDF 1921 kb) Additional file 4:
**Misclassification error rates as the classification threshold increases.** A .pdf file with a graph showing the average misclassification error rates of each implemented classifier as the classification threshold increases. (PDF 1856 kb) Additional file 5:
**List of genes with tissue-specific differential expression.** A.csv file with the list of genes that show tissue-specific differential expression in the five selected tissues as described in Methods. (CSV 30 kb) Additional file 6:
**Putative catalogue of synaptic genes.** A .csv file with the final catalogue of 893 *Drosophila* genes that according to the three algorithms have a probability above 90 % of being involved in synapse assembly and function. (CSV 35 kb) Additional file 7:
**Predicted probability of being synaptic for all genes in the data set.** A .csv file with the probability of being synaptic assigned by each of the three classifiers for all the genes in the data set. (CSV 573 kb) Additional file 8:
**Transcription profiles of synaptic genes.** A .pdf file with a graph showing the transcription profiles of the genes classified as synaptic at each threshold. (PDF 2061 kb)

## Abbreviations

CNS: Central nervous system
FPKM: Fragments per kilo base of exon per million fragments mapped
GO BP: Gene Ontology Biological Process
GO CC: Gene ontology cellular component
GO MF: Gene ontology molecular function
GTSDE: Genes with tissue-specific differential expression
k-NN: k - Nearest Neighbors
NGST: Next-generation sequencing technologies
RF: Random Forest
SVM: Support Vector Machine

## References

[1] Broadie K, Baumgartner S, Prokop A (2011). Extracellular matrix and its receptors in Drosophila neural development. Dev Neurobiol.

[2] Sigrist SJ, Schmitz D (2011). Structural and functional plasticity of the cytoplasmic active zone. Curr Opin Neurobiol.

[3] Frank CA, Wang X, Collins CA, Rodal AA, Yuan Q, Verstreken P, Dickman DK (2013). New approaches for studying synaptic development, function, and plasticity using *Drosophila* as a model system. J Neurosci Off J Soc Neurosci.

[4] Lassek M, Weingarten J, Volknandt W (2015). The synaptic proteome. Cell Tissue Res.

[5] Emes RD, Grant SGN (2012). Evolution of synapse complexity and diversity. Annu Rev Neurosci.

[6] Littleton JT, Ganetzky B. Ion channels and synaptic organization: analysis of the Drosophila genome. Neuron. 2000;26. 10.1016/s0896-6273(00)81135-6 10798390

[7] Burkhardt P (2015). The origin and evolution of synaptic proteins - choanoflagellates lead the way. J Exp Biol.

[8] Lloyd TE, Verstreken P, Ostrin EJ, Phillippi A, Lichtarge O, Bellen HJ (2000). A genome-wide search for synaptic vesicle cycle proteins in Drosophila. Neuron.

[9] Marcotte EM, Pellegrini M, Thompson MJ, Yeates TO, Eisenberg D (1999). A combined algorithm for genome-wide prediction of protein function. Nature.

[10] Schietgat L, Vens C, Struyf J, Blockeel H, Kocev D, Dzeroski S (2010). Predicting gene function using hierarchical multi-label decision tree ensembles. BMC Bioinformatics.

[11] Yan H, Venkatesan K, Beaver JE, Klitgord N, Yildirim MA, Hao T, et al. A genome-wide gene function prediction resource for *Drosophila melanogaster*. PLoS One. 2010;5. 10.1371/journal.pone.0012139 PMC2920829 20711346

[12] DeRisi JL, Iyer VR, Brown PO (1997). Exploring the metabolic and genetic control of gene expression on a genomic scale. Science.

[13] Eisen MB, Spellman PT, Brown PO, Botstein D (1998). Cluster analysis and display of genome-wide expression patterns. Proc Natl Acad Sci.

[14] Brown MP, Grundy WN, Lin D, Cristianini N, Sugnet CW, Furey TS, Ares MJ, Haussler D (2000). Knowledge-based analysis of microarray gene expression data by using support vector machines. Proc Natl Acad Sci U S A.

[15] Wen X, Fuhrman S, Michaels GS, Carr DB, Smith S, Barker JL, Somogyi R (1998). Large-scale temporal gene expression mapping of central nervous system development. Proc Natl Acad Sci U S A.

[16] Hvidsten TR, Komorowski J, Sandvik AK, Laegreid A (2001). Predicting gene function from gene expressions and ontologies. Pac Symp Biocomput Pac Symp Biocomput.

[17] Lukashin AV, Fuchs R (2001). Analysis of temporal gene expression profiles: clustering by simulated annealing and determining the optimal number of clusters. Bioinforma Oxf Engl.

[18] Lagreid A, Hvidsten TR, Midelfart H, Komorowski J, Sandvik AK (2003). Predicting gene ontology biological process from temporal gene expression patterns. Genome Res.

[19] Zhang W, Morris QD, Chang R, Shai O, Bakowski MA, Mitsakakis N, et al. The functional landscape of mouse gene expression. J Biol. 2004;3. 10.1186/jbiol16 PMC549719 15588312

[20] Lan H, Carson R, Provart NJ, Bonner AJ (2007). Combining classifiers to predict gene function in *Arabidopsis thaliana* using large-scale gene expression measurements. BMC Bioinformatics.

[21] Caruana R, Niculescu-Mizil A (2006). An empirical comparison of supervised learning algorithms. Proceedings of the 23rd international conference on Machine learning.

[22] Fernández-Delgado M, Cernadas E, Barro S, Amorim D (2014). Do we need hundreds of classifiers to solve real world classification problems?. J Mach Learn Res.

[23] Vinayagam A, Konig R, Moormann J, Schubert F, Eils R, Glatting K-H, Suhai S (2004). Applying support vector machines for gene ontology based gene function prediction. BMC Bioinformatics.

[24] Silverman B, Jones M (1989). E. Fix and J.L. Hodges (1951): an important contribution to nonparametric discriminant analysis and density estimation: commentary on Fix and Hodges (1951). Int Stat Rev Rev Int Stat.

[25] Breiman L (2001). Random forests. Mach Learn.

[26] Vapnik V (1998). Statistical learning theory.

[27] Prokop A, Meinertzhagen IA. Development and structure of synaptic contacts in Drosophila. Semin Cell Dev Biol. 2006;17. 10.1016/j.semcdb.2005.11.010 16384719

[28] Collins CA, DiAntonio A. Synaptic development: insights from *Drosophila*. Curr Opin Neurobiol. 2007;17. 10.1016/j.conb.2007.01.001 17229568

[29] Costello JC, Dalkilic MM, Beason SM, Gehlhausen JR, Patwardhan R, Middha S, et al. Gene networks in *Drosophila melanogaster*: integrating experimental data to predict gene function. Genome Biol. 2009;10. 10.1186/gb-2009-10-9-r97 PMC2768986 19758432

[30] Technau GM. Brain development in Drosophila melanogaster. Landes Bioscience, Austin, TX, and Springer Science+Business Media, New York. 2008.

[31] Marioni JC, Mason CE, Mane SM, Stephens M, Gilad Y (2008). RNA-seq: an assessment of technical reproducibility and comparison with gene expression arrays. Genome Res.

[32] Graveley BR, Brooks AN, Carlson JW, Duff MO, Landolin JM, Yang L, Artieri CG, van Baren MJ, Boley N, Booth BW, Brown JB, Cherbas L, Davis CA, Dobin A, Li R, Lin W, Malone JH, Mattiuzzo NR, Miller D, Sturgill D, Tuch BB, Zaleski C, Zhang D, Blanchette M, Dudoit S, Eads B, Green RE, Hammonds A, Jiang L, Kapranov P (2011). The developmental transcriptome of *Drosophila melanogaster*. Nature.

[33] Boley N, Wan KH, Bickel PJ, Celniker SE (2014). Navigating and mining modENCODE data. Methods San Diego Calif.

[34] Darbo E, Herrmann C, Lecuit T, Thieffry D, van Helden J. Transcriptional and epigenetic signatures of zygotic genome activation during early *Drosophila* embryogenesis. BMC Genomics. 2013;14. 10.1186/1471-2164-14-226 PMC3706223 23560912

[35] Krassovsky K, Henikoff S (2014). Distinct chromatin features characterize different classes of repeat sequences in *Drosophila melanogaster*. BMC Genomics.

[36] Tennessen JM, Bertagnolli NM, Evans J, Sieber MH, Cox J, Thummel CS (2014). Coordinated metabolic transitions during *Drosophila* embryogenesis and the onset of aerobic glycolysis. G3 Bethesda Md.

[37] Thomas JB, Wyman RJ (1984). Mutations altering synaptic connectivity between identified neurons in *Drosophila*. J Neurosci Off J Soc Neurosci.

[38] Kopczynski CC, Davis GW, Goodman CS (1996). A neural tetraspanin, encoded by late bloomer, that facilitates synapse formation. Science.

[39] Gorczyca M, Popova E, Jia XX, Budnik V (1999). The gene *mod(mdg4)* affects synapse specificity and structure in *Drosophila*. J Neurobiol.

[40] Wan HI, DiAntonio A, Fetter RD, Bergstrom K, Strauss R, Goodman CS (2000). Highwire regulates synaptic growth in *Drosophila*. Neuron.

[41] Featherstone DE, Broadie K (2000). Surprises from *Drosophila*: genetic mechanisms of synaptic development and plasticity. Brain Res Bull.

[42] Kraut R, Menon K, Zinn K (2001). A gain-of-function screen for genes controlling motor axon guidance and synaptogenesis in *Drosophila*. Curr Biol CB.

[43] Rieckhof GE, Yoshihara M, Guan Z, Littleton JT (2003). Presynaptic N-type calcium channels regulate synaptic growth. J Biol Chem.

[44] Long AA, Mahapatra CT, Woodruff EA, Rohrbough J, Leung H-T, Shino S, An L, Doerge RW, Metzstein MM, Pak WL, Broadie K (2010). The nonsense-mediated decay pathway maintains synapse architecture and synaptic vesicle cycle efficacy. J Cell Sci.

[45] Valakh V, Naylor SA, Berns DS, DiAntonio A (2012). A large-scale RNAi screen identifies functional classes of genes shaping synaptic development and maintenance. Dev Biol.

[46] Sieburth D, Ch’ng Q, Dybbs M, Tavazoie M, Kennedy S, Wang D, Dupuy D, Rual J-F, Hill DE, Vidal M, Ruvkun G, Kaplan JM (2005). Systematic analysis of genes required for synapse structure and function. Nature.

[47] Depner H, Lützkendorf J, Babkir HA, Sigrist SJ, Holt MG (2014). Differential centrifugation–based biochemical fractionation of the *Drosophila* adult CNS. Nat Protoc.

[48] Ashburner M, Ball CA, Blake JA, Botstein D, Butler H, Cherry JM, Davis AP, Dolinski K, Dwight SS, Eppig JT, Harris MA, Hill DP, Issel-Tarver L, Kasarskis A, Lewis S, Matese JC, Richardson JE, Ringwald M, Rubin GM, Sherlock G (2000). Gene ontology: tool for the unification of biology. The Gene Ontology Consortium. Nat Genet.

[49] Zhang W, Zhang Y, Zheng H, Zhang C, Xiong W, Olyarchuk JG, Walker M, Xu W, Zhao M, Zhao S, Zhou Z, Wei L (2007). SynDB: a Synapse protein DataBase based on synapse ontology. Nucleic Acids Res.

[50] Pirooznia M, Wang T, Avramopoulos D, Valle D, Thomas G, Huganir RL, Goes FS, Potash JB, Zandi PP (2012). SynaptomeDB: an ontology-based knowledgebase for synaptic genes. Bioinforma Oxf Engl.

[51] Lyne R, Smith R, Rutherford K, Wakeling M, Varley A, Guillier F, Janssens H, Ji W, Mclaren P, North P, Rana D, Riley T, Sullivan J, Watkins X, Woodbridge M, Lilley K, Russell S, Ashburner M, Mizuguchi K, Micklem G (2007). FlyMine: an integrated database for Drosophila and Anopheles genomics. Genome Biol.

[52] Lyne M, Smith RN, Lyne R, Aleksic J, Hu F, Kalderimis A, Stepan R, Micklem G (2013). metabolicMine: an integrated genomics, genetics and proteomics data warehouse for common metabolic disease research. Database.

[53] Wilhelm BG, Mandad S, Truckenbrodt S, Krohnert K, Schafer C, Rammner B, Koo SJ, Classen GA, Krauss M, Haucke V, Urlaub H, Rizzoli SO (2014). Composition of isolated synaptic boutons reveals the amounts of vesicle trafficking proteins. Science.

[54] Spellman PT, Rubin GM. Evidence for large domains of similarly expressed genes in the *Drosophila* genome. J Biol. 2002;1. 10.1186/1475-4924-1-5 PMC117248 12144710

[55] Hooper SD, Boue S, Krause R, Jensen LJ, Mason CE, Ghanim M, et al. Identification of tightly regulated groups of genes during *Drosophila melanogaster* embryogenesis. Mol Syst Biol. 2007;3. 10.1038/msb4100112 PMC1800352 17224916

[56] Papatsenko I, Levine M, Papatsenko D (2010). Temporal waves of coherent gene expression during *Drosophila* embryogenesis. Bioinforma Oxf Engl.

[57] Weber CC, Hurst LD. Support for multiple classes of local expression clusters in *Drosophila melanogaster*, but no evidence for gene order conservation. Genome Biol. 2011;12. 10.1186/gb-2011-12-3-r23 PMC3129673 21414197

[58] Bar-Joseph Z, Gitter A, Simon I (2012). Studying and modelling dynamic biological processes using time-series gene expression data. Nat Rev Genet.

[59] Cantera R, Ferreiro MJ, Aransay AM, Barrio R. Global gene expression shift during the transition from early neural development to late neuronal differentiation in *Drosophila melanogaster*. PLoS One. 2014;9. 10.1371/journal.pone.0097703 PMC4022633 24830291

[60] Adams MD, Celniker SE, Holt RA, Evans CA, Gocayne JD, Amanatides PG, Scherer SE, Li PW, Hoskins RA, Galle RF, George RA, Lewis SE, Richards S, Ashburner M, Henderson SN, Sutton GG, Wortman JR, Yandell MD, Zhang Q, Chen LX, Brandon RC, Rogers YH, Blazej RG, Champe M, Pfeiffer BD, Wan KH, Doyle C, Baxter EG, Helt G, Nelson CR (2000). The genome sequence of *Drosophila melanogaste*r. Science.

[61] McQuilton P, St Pierre SE, Thurmond J (2012). FlyBase 101--the basics of navigating FlyBase. Nucleic Acids Res.

[62] Zhang W, Zou S, Song J (2008). Term-tissue specific models for prediction of gene ontology biological processes using transcriptional profiles of aging in *Drosophila melanogaster*. BMC Bioinformatics.

[63] Mitsakakis N, Razak Z, Escobar M, Westwood JT (2013). Prediction of *Drosophila melanogaster* gene function using Support Vector Machines. BioData Min.

[64] Zhao X-M, Wang Y, Chen L, Aihara K (2008). Gene function prediction using labeled and unlabeled data. BMC Bioinformatics.

[65] Chintapalli VR, Wang J, Dow JAT (2007). Using FlyAtlas to identify better *Drosophila melanogaster* models of human disease. Nat Genet.

[66] Hastie T, Tibshirani R, Friedman JH (2009). The elements of statistical learning data mining, inference, and prediction.

[67] R Development Core Team: R Development Core Team (2013). R: A language and environment for statistical computing. R Foundation for Statistical Computing, Vienna, Austria. ISBN 3-900051-07-0, URL https://www.r-project.org/.

[68] Wiener M (2002). LA: Classification and regression by randomforest. R News.

[69] Leisch F, Weingessel A, Hornik K, Dimitriadou E, Meyer D (2012). e1071: Misc Functions of the Department of Statistics (e1071), TU Wien. R package version 1.6-1.

[70] Venables WN, Ripley BD (2002). Modern applied statistics with S.

[71] Eden E, Navon R, Steinfeld I, Lipson D, Yakhini Z (2009). GOrilla: a tool for discovery and visualization of enriched GO terms in ranked gene lists. BMC Bioinformatics.

[72] Gelbart WM, Emmert DB (2010). FlyBase high throughput expression pattern data beta version.

