# Supplementary material for: Putative synaptic genes defined from a Drosophila whole body developmental transcriptome by a machine learning approach
Source: BMC Genomics. 2015 Sep 15;16(1):694. doi: 10.1186/s12864-015-1888-3 (PMC4570697; doi:10.1186/s12864-015-1888-3)
Supplement: Additional file 3: — ROC curves of the three implemented classifiers. A .pdf file with the ROC curves of the three classifiers. (PDF 1921 kb) [file 12864_2015_1888_MOESM3_ESM.pdf]

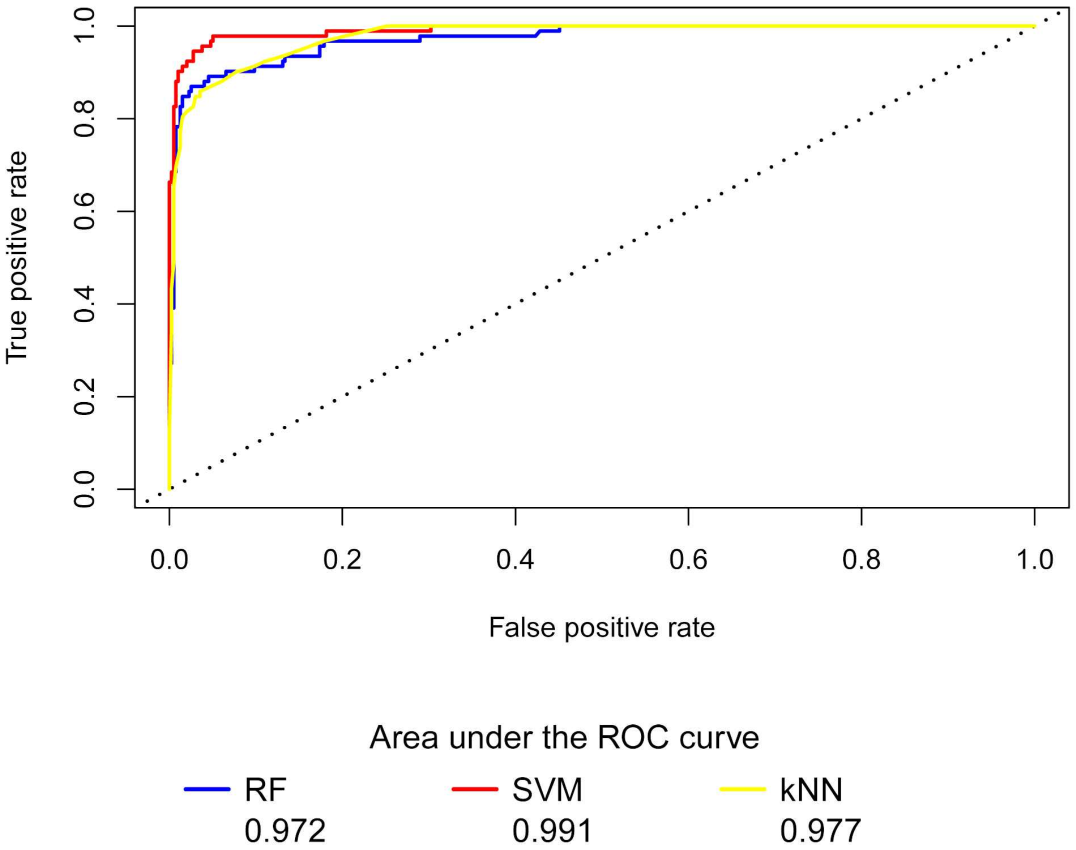

Additional file 3 - ROC curves of the three classifiers.
The graph shows the true positive error rate vs. the false positive rate for each classifier. These rates were caluclated in R. The area under the curve is shown under the graph.
