# Supplementary material for: Putative synaptic genes defined from a Drosophila whole body developmental transcriptome by a machine learning approach
Source: BMC Genomics. 2015 Sep 15;16(1):694. doi: 10.1186/s12864-015-1888-3 (PMC4570697; doi:10.1186/s12864-015-1888-3)
Supplement: Additional file 4: — Misclassification error rates as the classification threshold increases. A .pdf file with a graph showing the average misclassification error rates of each implemented classifier as the classification threshold increases. (PDF 1856 kb) [file 12864_2015_1888_MOESM4_ESM.pdf]

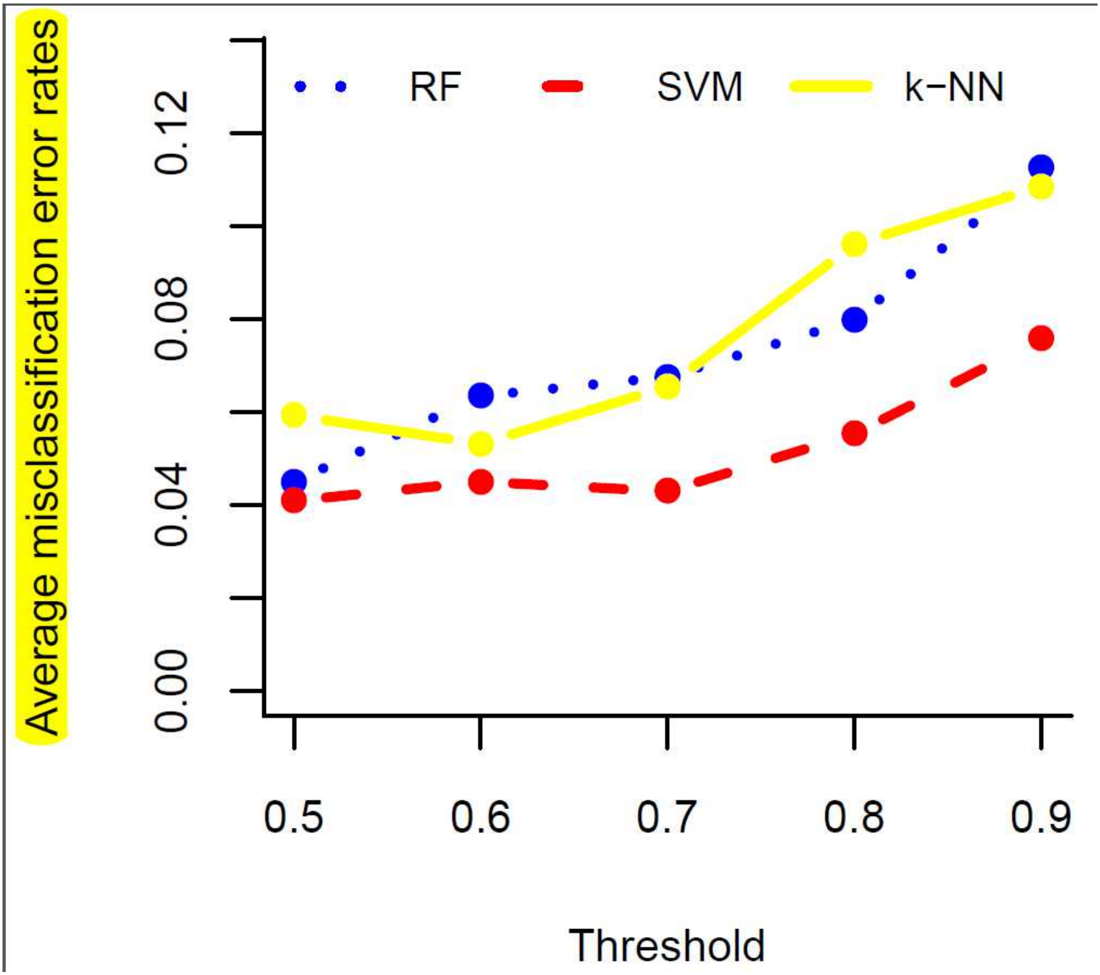

Additional file 4 - Misclassification error rates as the classification threshold increases. Average missclassification error rates were calculated, by ten fold cross validation, for each classifier and each classification threshold.
