# Supplementary material for: Putative synaptic genes defined from a Drosophila whole body developmental transcriptome by a machine learning approach
Source: BMC Genomics. 2015 Sep 15;16(1):694. doi: 10.1186/s12864-015-1888-3 (PMC4570697; doi:10.1186/s12864-015-1888-3)
Supplement: Additional file 8: — Transcription profiles of synaptic genes. A .pdf file with a graph showing the transcription profiles of the genes classified as synaptic at each threshold. (PDF 2061 kb) [file 12864_2015_1888_MOESM8_ESM.pdf]

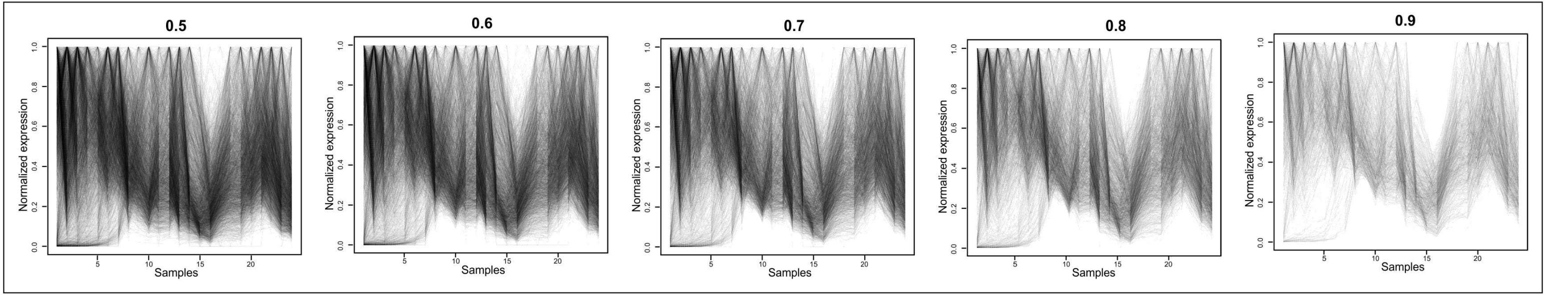

Additional file 8 - Transcription profiles of the genes classified as synaptic at each classification threshold.
Each graph shows the transcription profiles of the genes classified as synaptic by the three algorithms at each classification threshold. The corresponding classification threshold is shown above each graph, in which transcription profiles are shown in normalized FPKM values. The first graph corresponds to the initial classification done by the algorithms, and the last graph correspond to our final catalogue of putative synaptic genes.
